# Use Them for What They Are Good at: Mealworms in Circular Food Systems

**DOI:** 10.3390/insects12010040

**Published:** 2021-01-06

**Authors:** Hartmut Derler, Andrea Lienhard, Simon Berner, Monika Grasser, Alfred Posch, René Rehorska

**Affiliations:** 1 Institute of Applied Production Sciences, Sustainable Food Management, University of Applied Sciences FH JOANNEUM, Eggenberger Allee 11, 8020 Graz, Austria; andrea.lienhard@fh-joanneum.at (A.L.); simon.berner@fh-joanneum.at (S.B.); monika.grasser@fh-joanneum.at (M.G.); rene.rehorska@fh-joanneum.at (R.R.); 2 Institute of Systems Sciences, Innovation and Sustainability Research, University of Graz, Merangasse 18/1, 8010 Graz, Austria; alfred.posch@uni-graz.at

**Keywords:** *Tenebrio molitor*, circular food system, insect farming, by-products, circular economy

## Abstract

**Simple Summary:**

Different challenges exist, such as climate change and a growing number of people living on the planet, that put pressure on current and future food systems. In the future, more food must be produced on less land. At the same time, food-related greenhouse gas emissions must be reduced to be in line with the 2 °C climate goal, to limit potential risks of climate change. In this context, mealworms have been discussed as a sustainable and resource-efficient protein production option in circular food systems. They are an efficient biomass converter of low-quality by-products, such as wheat bran and brewer’s spent grain. In this article, we provide an overview of by-products that have been used in mealworm feeding trials. We quantify commonly available by-product types in Austria, and discuss potentials and limitations associated with mealworm farming. We found that further research is needed to better understand the strengths of mealworms in circular food systems, and several hurdles need to be addressed so that mealworm farming becomes more attractive in Western countries.

**Abstract:**

Future food systems must provide more food produced on less land with fewer greenhouse gas emissions if the goal is to keep planetary boundaries within safe zones. The valorisation of agricultural and industrial by-products by insects is an increasingly investigated strategy, because it can help to address resource scarcities and related environmental issues. Thus, insects for food and feed have gained increasing attention as a sustainable protein production strategy in circular food systems lately. In this article, we provide an overview on by-products, which have already been fed to *T. molitor* (mealworms), a common edible insect species. In addition, we investigate other by-products in Austria, which can be suitable substrates for *T. molitor* farming. We also provide an overview and discuss different perspectives on *T. molitor* and link it with the circular economy concept. We identify several future research fields, such as more comprehensive feeding trials with other by-products, feeding trials with mealworms over several generations, and the development of a standardized framework for insect rearing trials. In addition, we argue that due to their ability to convert organic by-products from agricultural and industrial processes into biomass in an efficient way, *T. molitor* can contribute towards resource-efficient and circular food and feed production. However, several hurdles, such as legal frameworks, need to be adapted, and further research is needed to fully reap the benefits of mealworm farming.

## 1. Introduction

The food system is a major contributor towards climate change and related environmental degradations [1]. Models indicate a significant increase in food-related greenhouse gas (GHG) emissions in the upcoming years, if countries such as China and India continue to adopt Western food consumption patterns [2]. Therefore, to keep the planetary boundaries within safe zones [3,4], future food systems must provide more food for more people on less land, and at the same time contribute towards carbon neutral societies. Thus, feeding more than 9 billion people in a sustainable way is a delicate endeavour, which involves profound changes within food systems. This already led Meyer–Rochow 45 years ago to suggest that insects could help to ease the problem of global food shortages [5]. In this context, sustainable intensification has gained increasing attention as a paradigm, in which sustainability and not productivity is the core strategy for agricultural development [6].

In a circular economy context, the valorisation of organic by-products from agri-food supply chains is a strategy to overcome resource scarcities. For instance, with about 10 million tonnes (MT) per year, the European Union (EU) is the largest wheat-producing region of the world, accounting for over 20 percent of worldwide wheat production. With a bran fraction between 25 to 30 percent, wheat generates a by-product stream of up to 3 MT per year. While it is used as animal feed, wheat bran is also often disposed of as waste [7].

Insects have gained increasing attention in the circular economy (CE) and sustainability debate, for the following reasons: (1) their ability to convert organic matter into protein more efficiently compared with their animal counterparts [8]; (2) in this respect, they can contribute in addressing the food waste and food loss problem [9,10,11]; (3) insect rearing involves less space, water, and often also less energy compared with their conventional livestock counterparts [12,13,14,15], and the environmental impact of insect-based products compared with animal-based products is lower [14,16]; (4) they have a nutrient profile similar to fish meat, so they can contribute to more balanced human and animal diets [17,18]; (5) they can serve as a substitute for ecologically critical substances, such as fishmeal and soymeal [19]. In addition, they can convert often unused and globally abundantly available organic residues, such as straw, food waste, green biomass, faeces, and manure into biomass efficiently [20,21,22]. In the light of these aspects, there is now considerable interest about the role of insects in circular food systems. Despite this interest, no one, as far as we know, has focussed on an analysis of national by-product streams, and their potential as substrates for *T. molitor* farming. In addition, insect farming has been rather loosely linked to the CE concept. We address this lack of clarity by stating recent literature on the CE and insects as food and feed domain.

Following that, we address two research questions in this article. First, which agricultural and industrial by-products have been fed to *T. molitor* and described in the literature? Second, what are promising fields of application of *T. molitor* in circular food systems? Thus, this article provides an overview and discusses current and future applications of *T. molitor* as a biomass converter in a CE.

The subsequent sections of this article are structured as follows. Section 2 gives a brief overview of the theoretical background of circular food systems and insects as sustainable protein sources, and states the applied method. Section 3 states the identified substrates. Section 4 continues with a discussion of the results, and integrates them into the broader insects and CE literature. Section 5 concludes the article by arguing that further research is needed to fully explore the potential of *T. molitor* in a circular food system.

## 2. Theoretical Background and Methods

### 2.1. Circular Economy and Insects

CE appeared first in the 1960s in Kenneth Boulding’s essay “The Economics of the Coming Spaceship Earth” [23]. Since then, many definitions of CE emerged [24]. Therein, the concepts “reduction”, “reuse”, and “recycling” are most frequently depicted, which encompass theories and principles from industrial ecology [24,25]. CE has received significant attention on the political and economic agenda, because of its potential to unlock economic growth in a sustainable way [26]. At the same time, the concept has received critique by scholars for being too vague. They state that it emerged as an unstructured combination of concepts from separate ideas and paradigms (e.g., industrial ecology, bioeconomy, cradle-to-cradle) and semi-scientific concepts [24]. In addition, they claim that links to the concept of sustainable development have not been drawn sufficiently [24,27,28]. Geissdoerfer et al. [29] investigated the relationship between the two concepts and highlight that CE should be “[…] viewed as a condition for sustainability, a beneficial relation, or a trade-off in literature”. Despite myriad definitions, critiques, and concepts linked to CE, throughout this paper we draw on the definition of Geissdoerfer et al. [29], who define “[…] Circular Economy as a regenerative system in which resource input and waste, emission, and energy leakage are minimised by slowing, closing, and narrowing material and energy loops”. We choose this definition because in our view, it represents a concise definition that fits with the discussed perspectives of insect farming and CE provided in this article.

In the light of contemporary social, ecological, and economic agricultural challenges, a growing body of literature has linked CE with the agri-food system research [30], and in particular with insect farming [31]. For instance, Cappellozza et al. [32] state a case study on a regenerating system, in which they fed black soldier flies (BSFs) with fruit and vegetable leftovers. The insect biomass was transformed into meal and oil feeding, and the residuals from rearing served as substrates to grow earthworms. Finally, a compost for plants was used to close the material loop. Barbi et al. [33] fed BSFs with agri-food leftovers, and found that the larvae development was favoured by a mixture of legume, corn, and fruit leftovers. Skrivervik [34] investigated the contribution of insect farms to the bioeconomy, and state that although until today, none of the insect farms in the private sector use food waste as feed, it can be expected that this will change when regulatory frameworks are adapted accordingly. Cadinu et al. [31] provide a short review on the circularity of insect rearing, and argue that insect farming is an advantageous choice within a CE.

De Boer and van Ittersum [35] state three principles of circular food production in Europe. First, plant biomass must be the basic building block of food, and therefore, should be used by humans first. Second, by-products from food production, processing, and consumption should be recycled back into the food system. Third, animals should be used “[…] for what they are good at” [35]. Referring to the latter two principles, insects are known to have low feed conversion ratios (FCRs), surpassing the FCR of livestock, and they have been demonstrated to grow on several agricultural and industrial by-products. For instance, Oonincx et al. [20] fed Argentinean cockroaches, BSFs, *T. molitor*, and house crickets on diets composed of food by-products, and found that on suitable diets all insects utilized protein more efficiently compared with their conventional livestock counterparts, and *T. molitor* and house crickets were as efficient in the conversion of feed into edible body mass as poultry (i.e., 2.3 for poultry meat vs. 1.8–2.2 for yellow mealworm vs. 2.3 for house crickets). These results indicate that insects are “good at” the valorisation of low-quality by-products into edible proteins.

In summary, the main reasons why insects are prominently discussed in the CE and agri-food system fields are that they can contribute to (a) the reduction of food waste and losses, and the targeted valorisation of both; (b) the reduction of emissions, such as greenhouse gases, in food production; and (c) a shift from a linear economic model of taking, making, using, and disposal toward a closed-loop, low-carbon economy [36].

### 2.2. Method

In order to investigate the potential of *T. molitor* in Austrian circular food systems, we proceeded as follows: first, we conducted a literature review on by-products that have been used as substrates to feed *T. molitor*. Second, we identified local by-products that exist in higher quantities in Austria—both by-products that have already been used in *T. molitor* feeding trials, as well as those that, to our knowledge, have not been used in *T. molitor* feeding trials (i.e., sugar beet tops, grape and apple pomace, pumpkin seed meal). Third, we quantified the by-product streams based on average production quantities from 2010 to 2019, according to national production statistics [37,38,39,40] and the known average share of by-products resulting from the food processing (e.g., wine, sugar) [41,42,43]. For cereal and sugar beets, we used the fruit-to-straw calculator by the Austrian Chamber of Agriculture [44]. Subsequently, we described the main features of the by-products, such as their current uses, their nutritional relevance as a feed source, and their storage capabilities [45,46].

## 3. Results

Table 1 lists the identified by-products from agriculture and food industry in Austria that are available to be utilized in circular food systems. We added several types of information to the table, such as rival uses, whether the feed source exists only in specific regions in Austria, or whether it needs to be further processed. The last column indicates the studies dealing with the effects of the mentioned by-product on the growth performance of *T. molitor*. The results of these studies will be discussed in Section 4.

### 3.1. Brewing

On average, 9.1 million hectolitres of beer are brewed in Austria every year. Brewer’s spent grain (BSG) represent 85% of the total by-products generated in the brewing process, with approximately 20 kg of BSG residues and about 2 kg brewer’s yeast per hectolitre of beer. Thus, every year about 182,000 tons of BSG and 18,200 tonnes of yeast represent beer by-products in Austria. Due to its high share of protein (19–30% weight per weight), BSG is used as animal feed. In addition, it is used in biogas sites and in the production of flakes and biscuits. While the by-products from brewery processes are available in every region in Austria, annual fluctuations can occur. BSG tends to be available in higher quantities during the summer, because warmer climate conditions favour beer consumption. As BSG is in danger of spoilage, it needs to be further processed. Silaging is the most frequently applied method to increase its shelf-life, because this process does not alter its nutritive value.

### 3.2. Sugar Beet

Sugar beet (*Beta vulgaris*, L.) is cultivated in the north-eastern part of Austria. In the past 10 years, between 2.15 and 4.2 million tons of sugar beet have been harvested. The beet root contains about 20% sugar. The two main by-products of sugar beet processing into sugar are beet pulp and beet molasses. Due to its high sugar share, the latter is commonly used as animal feed. Approximately 38 kg beet molasses occur per ton of processed sugar beet root. Thus, on average about 117,400 tons of molasses are produced in Austria per year. Alternative uses of sugar beet molasses are animal feed and bioethanol production. In addition, about 1.8 million tonnes of sugar beet tops are produced in Austria. Sugar beet tops can be made into silage and fed to ruminants. The by-products from sugar production occur only during fall and winter, when sugar production takes place. Therefore, to extend their shelf-life they need to be further treated (e.g., by ensiling).

### 3.3. Fruit

The largest fruit by-product streams in Austria stem from the production of wine and apple juice. Over the last 10 years, on average about 2.3 million hectolitres (hl) of wine were produced in Austria. Per hectolitre of wine, around 33.3 kg pomace occurs, which results in 76,590 tons of grape pomace per year. Due to extreme weather events and adverse climate conditions, the quantity of wine has fluctuated significantly over recent years in Austria, and thus also the availability of its by-products (i.e., 1.7 million hl were produced in 2010, whereas 2.8 million hl in 2018). A total of 59,092 hl of apple juice was produced on average in the past 10 years. Per hl apple juice, about 30 kg pomace accrues, resulting in an average production of 1,772 tons of pomace. Apple and wine pomace has a high moisture content. Thus, it is often ensiled or dehydrated to avoid spoilage and extend its shelf-life. The by-products from grape and apple have seasonal constraints, i.e., fall and winter, when local wine and apple juice production takes place.

### 3.4. By-Products of Cereal Crops and Oil Seeds

About 20 to 25 percent of the whole wheat grain are by-products, of which 50 percent is wheat bran. Based on an average production of 1,608,571 tonnes of wheat, every year about 178,730 tonnes of wheat bran are collected in Austria. Wheat bran is a by-product from dry milling and commonly used feedstuff. The wheat by-products are produced in Austria throughout the year, although the amount of wheat produced during winter is less (winter crops). About 1.1 million tonnes of wheat straw result from wheat cultivation (2 million tonnes, including barley, oats, and rye). Wheat straw must be further treated, such as through shredding and drying.

Rape, sunflowers, and pumpkins are the most commonly cultivated oil plants in Austria. They are mainly cultivated for edible oil and for the production of bioethanol. Geographic limitations exist in Austria. While pumpkins are mainly produced in the states of Styria and Lower Austria, rape and sunflower are mainly produced in the states Upper Austria, Lower Austria, and Burgenland. Press cakes are the by-product of oil pressing, which is usually processed to meal. Approximately 135,000 tonnes of meal residues result from oil seed pressing (i.e., pumpkin, rapeseed, and sunflower) in Austria. Due to its high protein share, press cakes are often used as a feed for ruminants, pigs and poultry.

### 3.5. Crop Failures and Other Substrates

For several reasons (e.g., droughts, insect infestations) the incidence of crop failures have increased in Austria lately. For instance, due to a *Curculionidae* infestation in 2018, the sugar beet cultivated area in Austria declined by 27%. A 70% potato crop failure was also recorded in this year, due to an exceptionally dry summer that favoured the occurrence of *Agriotes lineatus* (click beetle). Damaged or infested crop products are mainly used as inputs in biogas production, and are composted or disposed as wastes. Due to climate change and changing consumer demands, farmers increasingly cultivate other plants in Austria, such as sweet potatoes and soy. As such, from 2010 to 2019 soy production more than doubled from 94.543 to 215.277 tonnes. In the future, new by-products relevant for *T. molitor* rearing may arise from the cultivation and food processing of these plants.

## 4. Discussion

Several authors applied BSG as a substrate in feeding trials with *T. molitor*. Oonincx et al. [20] used BSG in a high protein, high fat diet, together with beer yeast and cookie remains (60:20:20 ratio). They found that the survival rate (79.0 ± 7.0%) and development time (116.0 ± 5.2 days) of the insect larvae were similar to that with the control diet. However, they noted that cinnamon was part of the cookie remains, and most likely had an adverse effect on the growth performance and development rate. Melis et al. [52] fed mealworms with a pure BSG diet, with vegetables as a water source, and found a similar FCR as Oonincx et al. [20] (4.352 ± 0.451 after 14.10 weeks vs. 4.500 ± 0.170). Melis et al. also reported that the performance of mealworms was better with BSG compared with a pure wheat bran diet. Mancini et al. [53] fed mealworms with BSG and a mixed BSG bread and cookie leftover diet. Compared with the mixed diet, the pure BSG diet resulted in a faster growth period. They reported an FCR of 2.22 for the BSG diet, and indicated that their results are comparable to those reported by van Broekhoven et al. [54] and Oonincx et al. [20]. In general, these studies indicate that BSG is a suitable substrate for the rearing of *T. molitor*.

Oonincx et al. [20] used beet molasses in a mixed diet together with (a) beer yeast and potato steam peelings (20:30:50) and (b) bread and potato steam peelings (20:30:50). Compared with the BSG, the mixed molasses diet was not so efficiently converted (4.5 ± 0.17 and 6.1 ± 0.62, respectively). Feeding trials with a pure beet molasses diet have not been described in literature.

In several feeding trials, wheat bran was used as a component of mixed diets with other by-products. Liu et al. [55] fed *T. molitor* with a wheat bran diet and supplemented fresh carrots, orange, and red cabbage to it. They stated that the supplementation accelerated the growth rate compared with the pure wheat bran diet (29.3% compared to a 37.9–49.3% weight increase). Feeding trials with wheat bran are comprehensively documented in Ribeiro et al. [47], who conducted a literature review about the optimal conditions for the mass rearing of *T. molitor*. They stated three core diets that were most frequently applied to *T. molitor*: first, a diet based on bran/flour added with a water source (i.e., vegetable, such as carrot rings or water); second, the same diet added with a protein source, such as soybean; and third, a varied artificial diet.

Several authors have stated the results of feeding trials with poor quality, wheat-based feedstocks. Yang et al. [48] fed mealworms with a combined wheat straw (WS) protein diet and found that, while having a high share of lignin, a WS-supplemented diet can support the growth of mealworms. After 32 days, the weights of mealworms fed with wheat bran was higher than with WS (6.18 mg ± 1.34% versus 2.54 mg ± 0.89%). Stull et al. [49] fed *T. molitor* with maize stover, and found that in a mixed diet some grain feedstock could be replaced by maize stover without hampering the nutrient content of the larvae. In a second experiment, they found that multiple generations of *T. molitor* could survive a pure stover diet. At the same time, the per-day growth rate of the pure stover diet was lower (2.38 vs. 3.65 mg per day) compared with the control diet (i.e., wheat bran, oats, brewer’s yeast; 50:45:5% by weight). Davis et al. [50] fed *T. molitor* with different rapeseed canola diets (i.e., Tower and Candle ground seeds, defatted ground hulls, defatted ground dehulled fractions; note that Tower and Candle are varieties of canola seeds (*Brassica campestris*)). After four weeks, the mealworms had weight gains ranging from 35.9 mg (dehulled Tower canola seed with 0.5 Candle hulls) to 54.6 mg (Tower ground seed). Compared with the per-day growth rate for maize stover (2.38 mg), the rapeseed canola diet resulted in smaller weight gains than the maize stover diet.

Zhang et al. [56] fed mealworms with a mushroom spent corn stover, and compared it with wheat bran as the control diet. After 60 days, the dry weights of mealworms reached 67% of the mealworms reared with wheat bran. They stated that although the growth performance was less favourable compared with the control diet, the nutritional profile of the larvae was just as good as or even superior. They stated that it might be commercially viable to use corn stover as a diet supplement, because the costs of the by-product are lower compared with wheat bran.

Ruschioni et al. [57] fed *T. molitor* with an olive pomace-enriched substrate based on wheat middlings (3:1, 1:1, and 1:3 ratios). They found that a 3:1 wheat middling pomace ratio was best with respect to the growth and nutritional performance of *T. molitor*. Increases in olive pomace had an adverse effect on the development time of the larvae. They stated that the growth performance in terms of mean larval development time was comparable to those described in van Broekhoven et al. [54].

A limitation that arises from these studies is that the feeding trials were conducted under different laboratory conditions, and different performance indicators were used (i.e., development time in days; survival rate; weight gains per day; FCR; weight gains after 30 or 60 days, etc.). Therefore, a guideline for standardised insect feeding trials could be elaborated, which would allow a better comparison of insect rearing studies.

In principle, crop failures and damaged vegetables and fruits are an interesting substrate source for *T. molitor* and insects, due to their vast availability. At the same time, a potential shortcoming of these plants are mycotoxins. In this respect, several publications have investigated the digestibility of infected vegetables and wheat, and their effects on the growth performance and mortality of the insects. Van Broekhoven et al. [58] and Ochoa Sanabria et al. [59] used deoxynivalenol-contaminated wheat (DON) as a substrate for *T. molitor*. While the first article reports no DON residuals, the latter found very low DON accumulation in the harvested larvae. Both articles suggest that the filamentous fungus *Fusarium*-contaminated wheat could be used to produce insect protein. Early research on the effect of different *Fusarium* species and on the growth performance of *T. molitor* suggest that fungal isolates result in growth depressions of the insect larvae [60]. Schrögel and Wätjen [61] conducted a literature review on substrate contamination with mycotoxins, and found no evidence for mycotoxin accumulation in *T. molitor*. However, they noted that the literature data on the metabolism pathways of mycotoxins in insects is still limited. Despite these first promising results with respect to the food safety of mealworms grown on *Fusarium*-contaminated feed, further research is needed, e.g., with respect to the enzymatic degradation of DON in *T. Molitor* and the toxicity of the resulting metabolites [58]. At the same time, Schrögel and Wätjen [61] suggest that, subject to a strict monitoring of contaminants, a general ban of food waste in insect farming in the EU should be questioned.

We identified several other substrates in the literature that have been used for mealworm rearing, but which are prohibited as insect or animal feed in Western countries due to food safety regulations. For instance, an interesting ability of *T. molitor* is that it can eat and possibly metabolise part of ingested polystyrene, polyvinyl chloride, low-density polyethylene, and styrofoam [62,63], and there is increasing evidence that polystyrene biodegradability is likely to be ubiquitous within the genus *Tenebrio* [63,64,65]. However, polystyrene-induced diets had adverse effects on the growth performance of the mealworms and increased their mortality compared with control diets. In addition, *T. molitor* and other insects, such as BSFs, are known to be efficient biomass converters of critical substrates, including animal and human faeces [66,67,68], and studies of fly larvae for organic waste treatments have shown their effective and economically feasible biodegradation [10,69,70]. Early research indicates that by-products from animal processing, such as blood and offal, can have positive impacts on the growth performance of mealworms [71]. Despite the vast availability of these substances, the general ban of these as animal feed is unlikely to change in the foreseeable future, because a vast number of studies would be needed to confirm their food safety.

Due to their high fat content, BSF and *T. molitor* can play an important role in biorefinery processes and in the production of biodiesel [72,73,74,75]. Also, Murugan et al. [76] have reported the possibility of applying mealworms as a biological agents to purify bacterial polyhydroxyalkanoates, and Houben et al. [77] state that *T. molitor* frass can serve as a substitute of mineral NPK (i.e., nitrogen, phosphorus, and potassium) fertilizer. These examples demonstrate the versatile potential applications of mealworms and insects in circular food systems.

The sustainability of insects as food and feed has been widely discussed for the last decade, and numerous arguments have been made in favour rearing insects on an industrial scale. A crucial factor for successful insect farming is that suitable substrates have to be found, which allow the resource- and cost-efficient mass rearing of insects. As such, these substrates have to fulfil numerous requirements, such as being free from any harmful contaminants. Furthermore, they should be locally available in large quantities and organic side-streams, logistically easy to handle, storable with a long shelf-life, and placed on the market with a competitive price. We have quantified and analysed several by-product streams in Austria, which can serve as substrates for *T. molitor* rearing. In this regard, many by-products have seasonal and local constraints. For instance, BSG tends to occur in higher amounts during the summer, when more beer is consumed. Bottlenecks of substrates can be overcome through the substitution of by-products that have a similar nutrient profiles. Besides, as most by-products must be dried before they are fed to *T. molitor*, seasonal constraints can be targeted through the curing of by-products. However, this could increase environmental and production costs of the by-products.

Hence, a critical issue of insect rearing in CE is its cost efficiency [31]. Roffeis et al. [78] conducted a life cycle cost assessment of insect-based feed production, and found that while insect feed showed cost disadvantages in relation to plant-based feed, the same does not hold for animal-based feeds such as fishmeal. Automatized modular systems, such as containers that are designed for a decentralized insect food waste treatment, close to the by-product origin (e.g., at breweries) may represent a cost-efficient solution to generate insect biomass. In this respective, Ites et al. [79] found that modular technologies can lead to more environmentally efficient and comparably economically efficient results as composting. Concerning the cost efficiency of insect mass rearing, Maillard et al. [80] analysed the organisation of middle- to very large-sized mealworm raising and processing sites of up to 10,000 tons per year. They state several hurdles for the establishment of large-scale insect farms, such as space occupancy, the need for automation technologies, and a stable supply to the market. According to them, these aspects need to be targeted such that insect protein can economically compete with plant-based protein.

Fibre-rich substrates, such as WS, may be added to the substrate mix to reduce feed-related costs. According to Zhang [56], it may be commercially viable to grow *T. molitor* on by-products, such as distillers’ grains and corn stover, because these by-products are less expensive compared to wheat bran. At the same time, many of these by-products must be processed prior to feeding (i.e., shredded, dried or ensilaged), potentially adding environmental and production costs to the final insect protein. In summation, further research concerning the cost-efficiency in this field is urgently needed.

According to Baiano [81], the non-standardised legal status of edible insects is a main hurdle that needs to be addressed on the global scale. Thus, in the future, the legal framework should be adapted such that insect farming becomes more attractive in the private sector. At the same time, the integration of the insect welfare considerations should be sufficiently addressed before propagating large-scale insect farms [82].

In Section 2, we state that in circular food systems, animals should be used for “what they are good at”. Previous research has shown that in circular food systems, insects can play a pivotal role by converting unused by-products of industrial and agricultural food process into compost and feed [83]. In general, the production of insect protein comes along with less land and energy compared to the production of traditional animal protein forms. Therefore, it can be considered as a renewable source of food energy [84]. This is also true for less developed countries. There is a growing interest into the integration of insects in circular food systems in less developed regions, as they can promote inclusive businesses for small-scale farmers [85] and can contribute towards a more secure food provision. Thus, Adegbeye et al. [86] consider insect farming as a sustainable agricultural production option that helps to alleviate greenhouse gasses and address nutrient recycling in emerging and transitional nations.

An important factor that decides the circularity of the insect substrate is the substrate’s geographical origin. In circular food systems, short food supply chains should be considered as an integrative feature. As such, in their concluding remark on a transition towards circular economy in the food system, Jurgilevich et al. [25] highlight that “[…] circular economy sustainability solutions include supporting local food supply chains with less waste, closing nutrient loops, pricing the true cost of resource consumption and losses in natural capital, and creating policy mechanisms to promote recovery and reduce loss of critical raw materials in particular”. Thus, locally available by-product streams should be preferred in mealworm rearing sites. For instance, in Austria, the south-eastern region is known for its pumpkin oil seed production, whereas rapeseed is cultivated in the northern parts of Austria. Mealworm farming should consider these geographical framework conditions to optimize circular production flows.

An increasingly investigated field of application of insects as animal feed are aquacultures [31]. Several studies have confirmed that the partial dietary replacement of fishmeal by mealworms on rainbow trout have positive effects on their growth performance, with minor negative effect on their fatty acid profiles [87,88]. As most aquacultures depend on fishmeal, insect proteins can represent a more sustainable feed option. Similar results have been reported in feeding trials with pigs [89] and chickens [90].

Nevertheless, from a sustainability point of view, high feed conversion efficiencies do not necessarily lead to sustainable, sound insect protein. In their conclusions, Lundy and Parrella [91] state that the potential of insects as a sustainable alternative to the global protein supply chain depends on the identification of regional scalable side-streams that are of high quality and at the same time, do not rival livestock production.

From a consumer perspective, several articles have investigated the willingness of European consumers to include insects in their diets [92,93,94]. A main barrier of entomophagy is insect food neophobia, which is common among European countries. The incorporation of insects into familiar food items and tasting sessions can help to reduce food neophobia [94,95]. A study of Verbeke [93] suggests that the integration of insects into diets is more likely for specific groups, such as young males who are less attached to meat and follow a sustainability-driven consumption lifestyle. In addition, informative seminars can help to reduce the disgust factor associated with insect-based products [96]. Further research is needed to better understand consumer’s feelings, attitudes, beliefs, and motivations to choose insect food products [97].

We identified several other fields for future research. First, we found that most feeding trials did not last longer than 100 days, and were not conducted over several *T. molitor* generations. In addition, a standardised procedure (e.g., a guideline) for insect rearing that allows a better performance comparison between by-product types and insect species has been missing until now. Second, several feeding trials with by-products used a mixed substrate and did not consider pure by-product diets. Currently, we are conducting feeding trials in which we address this issue. Third, contradicting literature exists for the relationship between the growth performance and protein content of the substrates. While Mancini et al. [53] state that higher protein contents lead to lower FCR values, this is not supported by Zhang et al. [56]. More research is needed to better understand the relationship between the protein content and growth performances of *T. molitor*.

## 5. Conclusions

Climate change and resource scarcity will be primary challenges in the future, because more food must be produced on less land to be able to feed more than 9 billion people. Therefore, lately, increasing attention has been on circular, resource-efficient strategies in agricultural processes, such as biochar, integrated farm management schemes, and insect farming [86]. This research addresses the latter domain and discusses the role of *T. molitor* in circular food systems. As such, we put forth the imperative to “use them for what they are good at”, in reference to one of the three pillars on a circular agricultural production in Europe, as stated by De Boer and van Ittersum [35]. The main findings of our study are that even though there is an increasing research activity on the by-product conversion by *T. molitor*, there is still a lack of clarity with respect to the suitability of various by-product types (e.g., oil press cakes, pomaces), and more feeding trials are needed to better understand the role of *T. molitor* and insects in CE.

Potential positive impacts of insects on the global food system have been discussed in the literature at length [98,99]. There is a general consensus in the literature that even though insects have a great capacity to convert organic matter into edible proteins, from an economic point of view insect protein production cannot compete with conventional plant-based protein yet [31]. This can change if framework conditions are adapted, and more evidence is provided for the private sector that edible insects like *T. molitor* are a safe and healthy food consumption option. From a sustainability point of view, careful appraisals must be conducted when it comes to the choice of optimal substrates, since the best insect diets in terms of insect growth rates do not always imply the most sustainable way of rearing insects [58].

Apart from commonly stated substrates in the literature, such as wheat bran and BSG, we identified several by-products, such as grape and apple pomace and damaged or spoiled crops, that have not been tested in feeding trials so far. Despite the vast availability of these substances in circular food systems, it is unlikely that these will play a significant role in commercial insect rearing in the foreseeable future, due to food safety regulations. Our article has two limitations. First, we only focussed on *T. molitor* and did not consider other insect species in our analysis. Second, we did not incorporate cost-efficiency calculations in our analysis. Further work needs to be done that addresses these dimensions.

Regardless of the hurdles, such as legal constraints and knowledge gaps, there is the broad consensus that insect production will increase in the near future. As Houben et al. [77] puts it: “[i]nsect production is expected to dramatically grow in the next few years due to the increasing need of finding alternative sources of protein.” However, this growth is more likely to occur in southern and central Africa and Southeast Asia, because in contrast to Western countries, these regions already have a significant market demand for edible insects [81]. In future, this could lead to a situation in which insects are produced in Western countries but consumed in other parts of the world.

## Figures and Tables

**Table 1:** Selected industrial and agricultural by-product streams in Austria.

By-Product	Source	t/Year	Main Component	Seasonal Constraints	Other Uses	Local Constraints	Further Treatment	Reference
Wheat bran	Milling	178,730	Carbohydrate	⊠	Animal feed Cereals Human consumption	☑	-	[47]
Brewer’s spent grain	Breweries	182,000	Protein Fibre	☑	Animal feed	⊠	Ensiling drying	[20]
Brewers’ yeast	Breweries	18,200	Protein	☑	Animal feed	⊠	Drying	[20]
Sugar beet molasses	Sugar production	117,400	Carbohydrate	☑	Animal feed	☑	Drying	[20]
Sugar beet tops	Sugar production	1,854,544	Fibre	☑	Biogas Animal feed	☑	Ensiling	-
Wheat straw (incl. wheat, barley, oats, rye)	Agriculture	1,126,000 (2,039,215)	Fibre	⊠	Animal feed Fuel Biogas	☑	Shredding	[48]
Maize stover including CCM	Agriculture	2,105,660	Fibre	☑	Animal feed	⊠	Shredding	[49]
Grape pomace	Vine production	76,590	Fibre	☑	Animal feed composting	☑	Ensiling drying	-
Apple pomace	Juice production	1,772	Fibre	☑	Animal feed composting Fuel	☑	Ensiling drying	-
Pumpkin oil seed meal	Oil production	10,930	Protein	☑	Animal feed	☑	Drying	-
Rapeseed meal	Oil production	89,554	Protein	⊠	Animal feed	☑	Drying	[50]
Sunflower meal	Oil production	34,547	Protein	☑	Animal feed	☑	Drying	[51]
Crop failures	Agriculture	-	Divers	⊠	Biogas	⊠	Drying	-

Abbreviation: CCM, corn crop mix.

## Data Availability

Publicly available datasets were analyzed in this study. Links can be found in the references [37,38,39].
